# Frequency of thoracic recurrence based on pathological features in patients with ovarian epithelial tumors in stage I versus higher stages

**DOI:** 10.1007/s11604-022-01374-y

**Published:** 2022-12-28

**Authors:** Hiroki Matsutani, Go Nakai, Satoe Fujiwara, Satoru Takahashi, Kazuhiro Yamamoto, Masahide Ohmichi, Keigo Osuga

**Affiliations:** 1 Department of Diagnostic Radiology, Osaka Medical and Pharmaceutical University, 2-7 Daigaku-Machi, Takatsuki, Osaka 569-8686 Japan; 2 Department of Obstetrics and Gynecology, Osaka Medical and Pharmaceutical University, 2-7 Daigaku-Machi, Takatsuki, Osaka 569-8686 Japan; 3 Department of Radiology, Takatsuki General Hospital, 1-3-13 Kosobecho, Takatsuki, Osaka 567-1192 Japan

**Keywords:** Ovarian cancer, Stage I, Chest recurrence, CT, Follow-up

## Abstract

**Purpose:**

The aim of this study was to clarify the frequency of thoracic recurrence and identify associated pathological features in postoperative patients with borderline or malignant ovarian epithelial tumors (BMOT) in stage I versus higher stages.

**Materials and methods:**

A total of 368 consecutive patients with a single primary BMOT were treated at our hospital. This study included the 217 patients with no residual disease on the first CT after standard treatment. The timing and pattern of recurrence on follow-up CT images with a scan range from chest to pelvis were evaluated retrospectively. Patient characteristics, tumor histology, and stage were recorded from electronic medical records.

**Results:**

After a median follow-up period of 48 months, recurrence was detected by CT in 9 patients in stage I (*n* = 159) and 15 in stage II/III (*n* = 58) (*p* = 0.0001). Thoracic recurrence was detected in four patients in stage I and four in stage II/III (*p* = 0.15). Abdominal recurrence was identified as a factor associated with thoracic recurrence (*P* < 0.001). Clear cell carcinomas accounted for three out of four thoracic recurrences in stage I and two out of four in stage II/III, and had the highest rates of thoracic recurrence (7.7% in stage I and 22.2% in stage II/III) among all histological types associated with thoracic recurrence. Among patients with recurrence, thoracic recurrence-free probability (*p* = 0.38), median abdominal recurrence-free interval (18 vs 16 months; *p* = 0.55) and thoracic recurrence-free interval (16.5 vs 23 months; *p* = 0.89) did not differ significantly between stage I and stage II/III.

**Conclusion:**

The frequency and timing of thoracic recurrence did not differ significantly in postoperative patients with BMOT in stage I versus stage II/III. Abdominal recurrence and a histological type of clear cell carcinoma were most often associated with thoracic recurrence in stage I.

## Introduction

Ovarian cancer ranked as the eighth most common cancer diagnosis and cause of cancer death in 2018, causing an estimated 184,000 to 295,000 deaths worldwide [1]. Ovarian cancer is treated by surgery to determine its histological type and pathological stage, followed by postoperative chemotherapy if necessary. Several studies showed that abdominopelvic recurrence usually precedes thoracic recurrence in postoperative patients with advanced ovarian cancer [2, 3]. However, specific recommendations regarding scan range and timing of computed tomography (CT) have never been established, even in the guidelines of the National Institutes of Health (NIH) [4] and the National Comprehensive Cancer Network (NCCN) [5], due to inadequate evidence regarding routine follow-up imaging studies for ovarian cancer patients. Although CT including the thoracic area is the standard imaging modality [3, 5], investigation of risk factors for thoracic metastasis can help optimize the use of cross-sectional chest imaging to reduce unnecessary chest radiation exposure for postoperative patients.

In stage I ovarian cancer, patients with grade 3 disease or clear cell carcinoma have a higher risk of recurrence [6], and tumor grade is the most powerful prognostic indicator [7]. Additionally, the response rate to platinum-based chemotherapy differs by histological type [8].

These facts suggest that histological subtype is a risk factor for thoracic recurrence in postoperative stage I patients. However, the association between pathological subtype and thoracic recurrence in postoperative patients with stage I ovarian epithelial tumors has never been described.

Also, the tumor markers (CA125 and CA19-9) play a clinically important role in surveillance of the recurrence of the ovarian cancer. The differences in preoperative tumor marker level by histological type has been described [9]. However, the diagnostic value of tumor marker depending on the histological type has not been investigated. Therefore, the aim of this study was to clarify the frequency of thoracic recurrence and identify associated pathological features in postoperative patients with stage I disease compared to those with more advanced disease. Also, the diagnostic ability of the tumor marker detecting the tumor recurrence was investigated.

## Materials and methods

### Patients

**Fig. 1:**
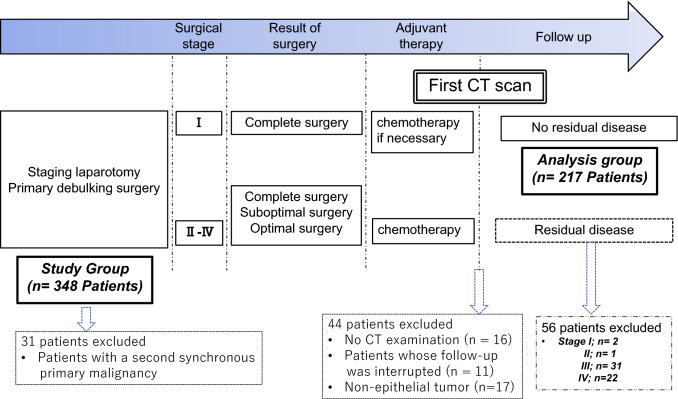
Flowchart showing ovarian cancer treatment process and patient selection

This retrospective study was approved by our institutional review board, and the requirement to obtain informed consent was waived. The subjects were selected from 348 consecutive patients with primary borderline ovarian epithelial tumors or carcinoma who underwent initial surgery at our hospital between June 2010 and February 2019. All patients who were candidates for optimal or complete surgery underwent staging laparotomy and debulking surgery. When primary debulking surgery was judged unfeasible, tumor stage was determined by laparoscopic biopsy, ascitic fluid cytology, or pleural fluid cytology as needed based on CT findings. When possible, interval debulking surgery was performed after neoadjuvant chemotherapy. The pathological stage was determined by a gynecological pathologist at our hospital. Postoperative chemotherapy was performed depending on postoperative results. All patients were exclusively followed by gynecologists at our hospital, and were referred for CT with a scan range from chest to pelvis as part of routine follow-up. Follow-up CT was performed every 1 year, 6 months, and 4 months in patients with stage I, stage II/III, and relapsed tumors, respectively. The pathology reports and electronic medical records of identified patients were reviewed to confirm the diagnosis and screen for eligibility. Patients who never underwent CT (*n* = 16), patients with a synchronous primary malignancy (*n* = 27), patients whose follow-up was interrupted (*n* = 11), patients with non-epithelial tumor (*n* = 17) and patients with residual disease on the first CT after standard treatment (stage I; *n* = 2, stage II; *n* = 1, stage III; *n* = 31, and stage IV; *n* = 22) were excluded, leaving 217 patients included for analysis. Figure 1 shows the ovarian cancer treatment protocol and patient selection.

### Data analysis

Contrast-enhanced CT using an intravenously administered non-ionic iodine-containing contrast agent was performed unless contraindicated. All axial CT images were acquired from 0.5-mm collimation reconstituted with a slice thickness of 2 mm, and were interpreted by two radiologists (H.M. and G.N.; 4 and 16 years of experience as radiologists, respectively) who were unaware of the patient’s cancer antigen (CA)-125 and CA19-9 levels, the International Federation of Gynecology and Obstetrics (FIGO) stages, and histological results, to assess for subtle recurrence.

Our criteria for CT features of recurrence were as follows: (a) a lesion that grows over time irrespective of adjuvant chemotherapy, (b) a lesion that decreases in volume after chemotherapy, (c) increasing unilateral pleural effusion with a positive cytology result, and (d) lymphadenopathy of cardiophrenic lymph nodes with a short axis ≥ 5 mm [10] or of other lymph nodes with a short axis ≥ 1 cm in diameter. Transient consolidation and interstitial changes in the lung fields were regarded as inflammatory changes. Lesions that did not progress on follow-up CT were deemed benign.

A consensus reading was performed for the case in which the two radiologists’ opinions differed. Multiplanar images were reviewed whenever axial images were insufficient to confirm whether the lesion should be regarded as a recurrence. Then, findings of each CT scan with a range from chest to pelvis, from the first scan after the standard treatment to the last scan, were analyzed for each patient.

Metastases in anatomic structures between the clavicles and the diaphragm, including pleural, pulmonary, nodal, thoracic soft tissue, and thoracic spinal metastases, were considered “thoracic recurrence.” Metastases involving the structures from the dome of the diaphragm inferiorly, including metastases within the abdominal cavity, wall, and lumbar spine, were defined as “abdominal recurrence.”

Tumor markers were measured at each visit, within 2 weeks of CT. Tumor marker elevation was defined as three consecutive increases [11] in either or both tumor markers. Patients with borderline tumors were excluded from this analysis.

The following were compared between patients in stage I and those in stage II/III: (1) metastatic sites (thoracic and/or abdominopelvic recurrence), their sequence over time, (2) patient and tumor characteristics associated with recurrence at any site, including thoracic recurrence, and (3) recurrence-free intervals and the interval between thoracic and abdominopelvic recurrence. Lastly, accuracy of tumor markers for detecting recurrence was compared between histological types.

### Statistical analysis

Associations of patient and tumor characteristics with overall recurrence and thoracic metastases were analyzed by the chi-square test. These included associations with tumor histological type, stage (I vs II/III), tumor markers, and presence of abdominal recurrence. CT interval (follow-up interval / number of CT scans performed), median abdominal recurrence-free interval (from primary surgery or laparoscopic staging in advanced cases to the first abdominopelvic recurrence), thoracic recurrence-free interval (from primary surgery or laparoscopic staging in advanced cases to the first thoracic recurrence) and interval between thoracic and abdominopelvic recurrence (from the first abdominal recurrence to the first thoracic recurrence) were compared using the Wilcoxon rank-sum test. Kaplan–Meier curves were constructed to confirm the effects of factors found to be associated with thoracic recurrence on the thoracic recurrence-free interval. A two-sided *p* value of less than 0.05 was considered to indicate a statistically significant difference. JMP® Pro 15.1.0 was used to conduct all statistical analyses.

## Results

### Patient characteristics and distribution of tumor histology (Table 1)

**Table 1:** Patient characteristics, tumor histology, and follow-up interval

Age (years), mean ± SD	53 ± 12.0 (range; 25–80)	
Stage		
I	159	
II	17	
III	41	
Histology		
Stage I		
HGSC	7 (4.4%)	
Mucinous carcinoma	14 (8.8%)	
Endometrioid carcinoma	28 (17.6%)	
Clear cell carcinoma	39 (24.5%)	
Borderline tumor	68 (42.8%)	
Mixed cell adenocarcinoma	3 (1.9%)	
Stage II/III		
HGSC	28 (48.3%)	
Mucinous carcinoma	0 (0%)	
Endometrioid carcinoma	15 (25.9%)	
Clear cell carcinoma	9 (15.5%)	
Borderline tumor	5 (8.6%)	
Undifferentiated carcinoma	1 (1.7%)	

Median CT interval in patients without recurrence (months)		*P* value*
Stage I (*n* = 159)	10.9, IQR 7.7–18.7	0.0001
Stage II/III (*n* = 58)	7.8, IQR 5.8–9.1	
Median CT interval in patients with recurrence (months)		
Stage I (*n* = 9)	4.1, IQR 3.1–6.5	0.47
Stage II + III (*n* = 15)	4.5, IQR 3.2–9.0	

*SD* standard deviation, *IQR* interquartile range, *HGSC* high-grade serous carcinoma

*Wilcoxon rank-sum test

The average age at diagnosis was 53 ± 12 years. The FIGO stage was I in 159 patients, II in 17 patients, and III in 41 patients. The histological types of stage I tumors were high-grade serous carcinoma (HGSC) (*n* = 7), mucinous carcinoma (*n* = 14), endometrioid carcinoma (*n* = 28), clear cell carcinoma (*n* = 39), borderline tumor (*n* = 68), and mixed cell adenocarcinoma (*n* = 3). Those of stage II/III tumors were HGSC (*n* = 28), mucinous carcinoma (*n* = 0), endometrioid carcinoma (*n* = 15), clear cell carcinoma (*n* = 9), borderline tumor (*n* = 5), and undifferentiated carcinoma (*n* = 1). Radiologic follow-up periods ranged between 4 and 109 months, with a median period of 47 months (interquartile range; IQR, 23.5–74.5). Median CT follow-up intervals for patients in stage I and stage II/III were 47.5 (IQR, 23–75.2) and 44 (IQR, 24.3–60.1) months, respectively, with no significant difference (*p* = 0.52). Median CT intervals in patients without recurrence were 10.9 (IQR, 7.7–18.7) months for those in stage I and a significantly shorter 7.8 (IQR, 5.8–9.1) months for those in stage II/III (*p* = 0.0001). In contrast, CT intervals in patients with recurrence did not differ significantly between stage I and stage II/III (4.1 (IQR, 3.1–6.5) and 4.5 (IQR, 3.2–9.0) months; *p* = 0.47).

### Metastatic sites and their sequence over time

**Fig. 2:**
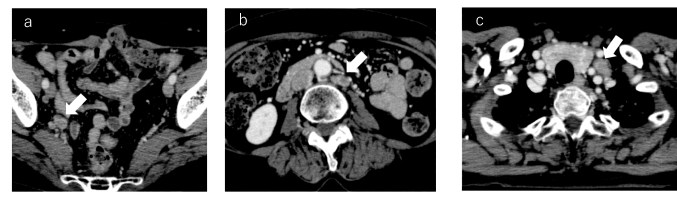
Contrast-enhanced CT images from a 60-year-old woman with stage IC clear cell carcinoma. **a** Right internal iliac lymph node metastasis (arrow) was detected 14 months after surgery. **b** Subsequently, para-aortic lymph node metastasis (arrow) and **c** left supraclavicular lymph node metastasis (arrow) were found 36 months after surgery

**Fig. 3:**
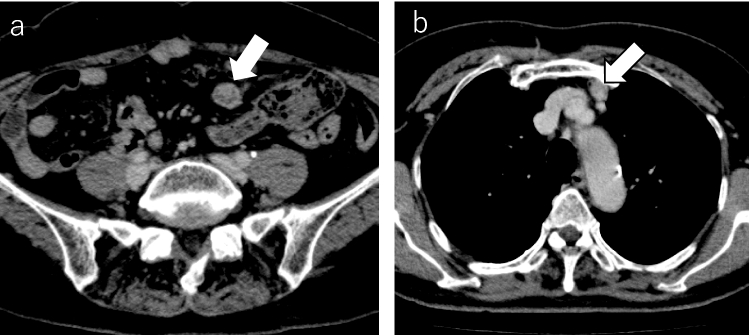
Contrast-enhanced CT images from a 68-year-old woman with stage IIIC HGSC. **a** Peritoneal dissemination (arrow) and **b** parasternal lymph node (arrow) metastasis were detected simultaneously at 16 months after surgery

Twenty-four patients (11% of all) had recurrence (stage I: *n* = 9, 5.2%; stage II/III: *n* = 15, 23.4%). Sixteen had abdominopelvic recurrence alone (stage I: *n* = 5, stage II/III: *n* = 11), and eight had both chest and abdominopelvic recurrence (stage I: *n* = 4, stage II/III: *n* = 4). All these eight patients regarded to have recurrence in this study were treated as having recurrence in practice. In these eight patients, abdominopelvic recurrence preceded thoracic recurrence or occurred simultaneously (Figs. 2, 3). No patient had only chest recurrence (Table 2). The forms of abdominopelvic recurrence were lymph node metastasis (*n* = 8) and peritoneal seeding (*n* = 17), including one patient with both forms. The forms of thoracic recurrence were lymph node metastasis, specifically to the cardiophrenic lymph nodes (CPLN) (*n* = 1), left supraclavicular lymph nodes (SLN) (*n* = 3) (Fig. 2), and parasternal lymph nodes (PSLN) (*n* = 1) (Fig. 3), as well as pleural dissemination in the form of pleural nodules (*n* = 2) or pleural effusion (*n* = 1), and finally pulmonary metastasis (*n* = 1). These figures include two patients who each had two forms of metastasis (pleural nodules plus pleural effusion in one patient and CPLN plus SLN in one patient). The pleural effusion was proven to be malignant by cytology.

**Table 2:** Patient and tumor characteristics associated with recurrence at any site and thoracic recurrence in postoperative patients with ovarian epithelial tumors

	*N*	Recurrence (*n* = 24)		*P* value*	Thoracic recurrence present (*n* = 8)		*P* value*
Age		58 ± 10.4			57 ± 8.7		
Stage		I	II/III		I	II/III	
		9	15	0.0001	4	4	0.15
Disease on abdominal imaging							< 0.001^§^
Absent	193	0	0		0	0	
Present	24	9	15		4	4	
Histology							
HGSC	35	2	7		0	2	
Mucinous carcinoma	14	1	0		0	0	
Endometrioid carcinoma	43	1	1		1	0	
Clear cell carcinoma	48	3	4		3	2	
Borderline tumor	73	1	2		0	0	
Mixed cell adenocarcinoma	3	1	0		0	0	
Undifferentiated carcinoma	1	0	1		0	0	

*N* number, *HGSC* high-grade serous carcinoma

*Chi-square test

^§^Association between presence of disease on abdominal imaging and thoracic recurrence

### Patient and tumor characteristics associated with recurrence at any site, including thoracic recurrence (Table 2)

**Fig. 4:**
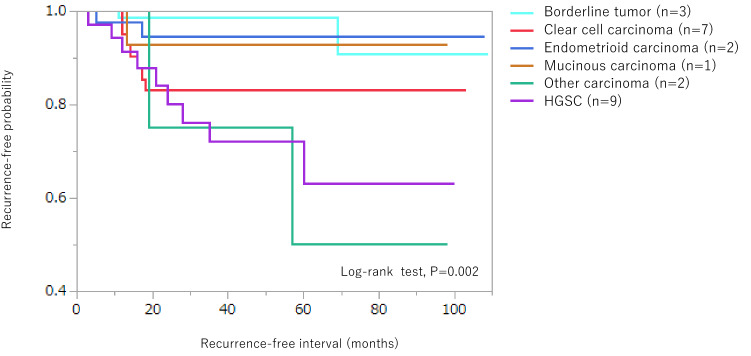
Kaplan–Meier curves showing differences in recurrence-free probability for all histological types. Recurrences were observed in all histological types. This indicates that frequency of recurrence and recurrence-free intervals vary widely for each histological type (log-rank test, *p* = 0.002). HGSC: high-grade serous carcinoma. Other carcinoma: mixed cell adenocarcinoma (*n* = 1) and undifferentiated carcinoma (*n* = 1)

The overall recurrence rate differed significantly between stage I and II/III (5.2% vs 23.4%; *p* = 0.0001). The breakdown of histological types in patients with recurrence at any site was as follows: HGSC (*n* = 9), mucinous carcinoma (*n* = 1), endometrioid carcinoma (*n* = 2), clear cell carcinoma (*n* = 7), borderline tumor (*n* = 3), mixed cell adenocarcinoma (*n* = 1) and undifferentiated carcinoma (*n* = 1). Histological type was significantly associated with recurrence-free probability (log-rank test, *p* = 0.002) (Fig. 4).

**Fig. 5:**
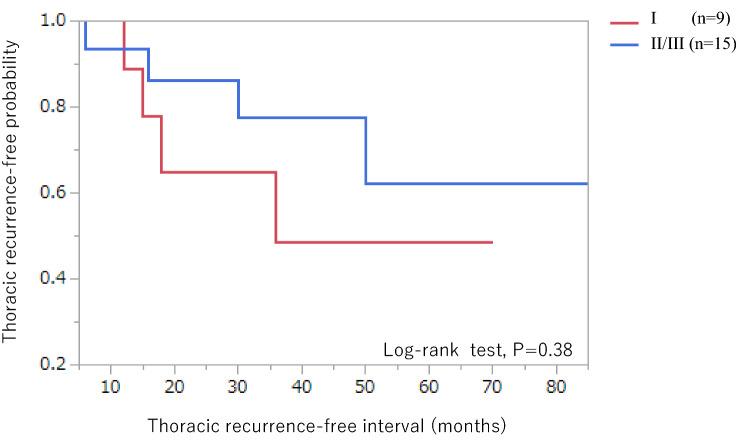
Kaplan–Meier curves showing differences in thoracic recurrence-free probability among patients with recurrence, in stage I versus stage II/III. The frequency of thoracic metastasis did not differ between stage I and stage II/III. (log-rank test, *p* = 0.38)

Four patients in stage I and four patients in stage II/III had thoracic metastases. The frequency of thoracic metastases did not differ significantly between stage I and stage II/III (*p* = 0.15). Among patients with recurrence, thoracic recurrence-free probability did not differ between stage I and stage II/III (log-rank test, *p* = 0.38) (Fig. 5). The breakdown of histological types in patients with thoracic metastases was as follows: HGSC (*n* = 2), endometrioid carcinoma (*n* = 1), and clear cell carcinoma (*n* = 5). Thoracic metastases were significantly associated with presence of abdominal recurrence (*P* < 0.001).

### Association between histological type and thoracic recurrence in stage I patients versus stage II/III patients (Table 2)

Clear cell carcinomas accounted for three out of four thoracic metastases in stage I and two out of four in stage II/III. Thoracic metastatic rates (the number of patients with thoracic metastases divided by the total number of patients in each group) of clear cell carcinoma, HGSC and endometrioid carcinoma in stage I were 7.7% (3/39), 0% (0/7), and 3.6% (1/28), respectively, and those in stage II/III were 22.2% (2/9), 7.1% (2/28), and 0% (0/15).

### Abdominal and thoracic recurrence-free intervals and the interval between thoracic and abdominopelvic recurrence (Table 3)

**Table 3:** Abdominal and thoracic recurrence-free intervals and the interval between thoracic and abdominopelvic recurrence

	Median (months)		*P* value*
	I (*n* = 9)	II/III (*n* = 15)	
Abdominal recurrence-free interval (*n* = 24)	18 (IQR 12.5–46)	16 (IQR 12–24)	0.55
	I (*n* = 4)	II/III (*n* = 4)	
Thoracic recurrence-free interval (*n* = 8)	16.5 (IQR 12.8–31.5)	23 (IQR 8.5–45)	0.89
Interval between thoracic and abdominopelvic recurrence (*n* = 8)	5 (range 0–22)	10.5 (range 0–37)	0.66

*IQR* interquartile range

*Wilcoxon rank-sum test

Median abdominal recurrence-free intervals in patients with stage I and those with stage II/III were 18 (IQR 12.5–46) and 16 (IQR 12–24) months, respectively. Corresponding thoracic recurrence-free intervals were 16.5 (IQR 12.8–31.5) and 23 (IQR 8.5–45) months, respectively. Median intervals between abdominal and thoracic metastases were 5 (range 0–22) and 10.5 (range 0–37), respectively. No significant differences in the abdominal recurrence-free interval, the thoracic recurrence-free interval, or the interval between abdominal and thoracic metastases were observed between patients with stage I and those with stage II/III (p = 0.55, 0.89, and 0.66, respectively).

### Accuracy of tumor markers for detecting recurrence by histological type of ovarian cancer

The sensitivity of tumor markers for detecting recurrence was 76.1% (16/21), specificity was 94.3% (116/123), PPV was 69.6% (16/23), NPV was 95.9% (116/121), and accuracy was 91.7% (132/144). Table 4 shows median tumor marker levels before surgery and those at the time of recurrence, as well as their sensitivity, specificity, PPV and NPV for detecting recurrence by each histological type. High specificity (92.3–96.2%) and NPV (92.3–97.5%) were observed regardless of histological type. There were no significant differences in median levels of both CA-125 and CA 19–9 before surgery or at the time of recurrence among all histological types.

**Table 4:** Median tumor marker levels preoperatively and at the time of recurrence, and the diagnostic ability of tumor markers for detecting recurrence by histological type

	Pre-CA125 (U/ml) [range]	Pre-CA19-9 (U/ml) [range]	CA125 (U/ml) [range]	CA19-9 (U/ml) [range]	Sensitivity (%)	Specificity (%)	PPV (%)	NPV (%)
HGSC (*n* = 9)	351.2 [8.9–1190]	18 [0.6–83.9]	21.8 [4.2–243.1]	8.4 [0.6–23.2]	77.8	96.2	87.5	92.6
Mucinous carcinoma (*n* = 1)	35.8	0.6	16	0.9	0	92.3	0	92.3
Endometrioid carcinoma (*n* = 2)	760 [85.9–1434]	425 [9.9–839]	610.9 [8.8–1213]	4.9 [1.3–8.4]	50	92.3	25	92.3
Clear cell carcinoma (*n *= 7)	153.7 [13.2–535]	36.7 [14.4–167]	43.8 [9.1–1678]	13.6 [5.5–64.6]	85.7	95.1	75	97.5
*p* Value*	0.21	0.14	0.67	0.14	–	–	–	–

*Pre-* preoperative, *HGSC* high-grade serous carcinoma, *PPV* positive predictive value, *NPV* negative predictive value

*Kruskal–Wallis test

## Discussion

This study revealed that the frequency of thoracic recurrence did not differ significantly between patients with stage I disease and those with stage II/III who had no residual disease on the first CT after standard treatment. Abdominopelvic recurrence always preceded thoracic recurrence or occurred simultaneously and the abdominal and thoracic recurrence-free interval also did not differ significantly between them. The histological type of clear cell carcinoma seemed to be more often associated with thoracic recurrence in stage I than stage II/III. Tumor marker levels at the time of recurrence did not differ between histological types and showed high specificity and NPV to detect recurrence regardless of histological type for all stages.

This study included more patients in stage I than those in more advanced stages (II/III), because patients who had residual disease on the first CT scan even after undergoing adjuvant chemotherapy had to be excluded to properly evaluate recurrence. Patients in stages III and IV were particularly likely to have residual disease, and were thus more often excluded from the study than patients in stage I (*n* = 53 vs *n* = 2). This affected the distribution of histological types between stage I and stage II/III. HGSC, which is known to have clinically aggressive behavior and relatively high chemo-sensitivity [8], was more common in stage II/III (*n* = 28) than in stage I (*n* = 7), despite the exclusion of more patients in stage II/III than those in stage I, while clear cell carcinoma is more often diagnosed in stage I than in a higher stage [8, 12, 13], and has a poorer overall prognosis and more chemotherapy-resistant behavior than HGSC and endometrioid carcinoma [8, 13, 14]. In this study, clear cell carcinoma was more common in stage I (*n* = 39) than in stage II/II (*n* = 9). At all stages, HGSC, which is commonly seen in stage II/III, showed a higher recurrence rate than clear cell carcinoma, which is commonly found in stage I (25.7% vs 14.6%). However, the thoracic recurrence rate of HGSC was lower than that of clear cell carcinoma (5.7% vs 10.4%). A study on pulmonary metastases in ovarian cancer published in 1985 before the introduction of cisplatin [15] showed that the incidence of metastasis did not correlate with tumor histological features. These results suggest that the more chemotherapy-resistant behavior of clear cell carcinoma may explain its greater incidence of thoracic recurrence despite its lower recurrence rate than HGSC.

The median CT interval in patients without recurrence was consistent with the typical CT interval for postoperative ovarian tumors in our hospital. Median CT interval in stage II/III (7.8 months) was significantly shorter than that in stage I (11 months). The difference in CT interval may have influenced the abdominal recurrence-free interval in this study. However, according to previous reports, patients with high-risk stage I ovarian cancer developed recurrence at a median interval of 18–22 months from diagnosis [6, 16] and those with advanced cancer developed recurrence at a median interval of 18–21 months [17, 18]. Our results regarding the median abdominal recurrence-free interval (18 months for stage I, 16 months for stage II/III) agree with their reports. Therefore, we believe that the difference in CT intervals had minimal influence on abdominal recurrence-free survival. In addition, the median CT interval in patients with recurrence was consistent with the typical CT interval used for patients with recurrence. The CT interval did not differ significantly between stage I and stage II/III. Consequently, CT follow-up including chest CT with the typical shorter interval (about 4 months) may be recommended for any patient with recurrence, regardless of stage, considering that the frequency of thoracic recurrence and intervals between thoracic and abdominopelvic recurrence did not differ significantly different between stage I and stage II/III.

All the first metastatic sites in this study were located at least in the abdomen or pelvis, and thus were visible on abdominopelvic CT. This implies a reduced need to perform routine chest CT for postoperative ovarian cancer, and agrees with previous studies indicating that pulmonary metastases tend to be preceded by abdominopelvic disease [15, 19, 20] and that all first thoracoabdominal metastases were visible on abdominal images [3].

The tumor markers CA-125 and/or CA19-9 are used as an indicator of ovarian cancer recurrence and those values did not differ significantly between histological types. Tanaka. Y.O. et al. revealed that preoperative elevation of CA-125 and CA 19–9 is associated with all types of cancer, including serous carcinoma, mucinous carcinoma, clear cell carcinoma, and endometrioid carcinoma. The level of CA19-9 was particularly high in mucinous carcinoma, but CA-125 did not differ between histological types [9]. Our results agreed with theirs, other than the CA19-9 level in mucinous carcinoma, probably because we only evaluated tumor markers in patients with recurrence. The preoperative CA-125 level is lower in clear cell carcinoma than HGSC [21], probably due to early manifestation [22]. Our results also showed elevated median postoperative CA-125 level in clear cell carcinoma at the time of recurrence. The elevated postoperative CA-125 in clear cell carcinoma was reported to be an independent risk factor for recurrence and survival [23] which might be associated with the highest rate of thoracic recurrence in this study.

The present study has two limitations. First, most of the positive findings of recurrence were based on our CT imaging criteria, not pathological examination. However, in clinical practice, chemotherapy is started when CT findings indicate distant metastasis in order to avoid an invasive biopsy to obtain a histological specimen of a recurrent lesion. Second, the study was a single-center retrospective study in Japan. Clear cell carcinoma is much more common in Japan than in Western countries, where HGSC accounts for the largest portion of tumors [24]. These differences in histological distribution may affect the frequency of thoracic recurrence in individual countries. Although thoracic recurrence-free probability did not differ significantly between clear cell carcinoma and the other histological types (HGSC or endometrioid carcinoma) associated with thoracic recurrences, the small number of patients with thoracic recurrences might affect *p* value in the log-rank test. Therefore, larger multicenter studies are needed to support our findings.

In conclusion, the frequency and timing of thoracic metastases in postoperative patients with stage I ovarian epithelial tumors did not differ significantly from those in patients with stage II/III tumors, although stage was significantly associated with recurrence. Presence of abdominal recurrence and a histological type of clear cell carcinoma were most strongly associated with thoracic metastases in stage I.
